# *In vivo* label-free photoacoustic flow cytography and on-the-spot laser killing of single circulating melanoma cells

**DOI:** 10.1038/srep39616

**Published:** 2016-12-21

**Authors:** Yun He, Lidai Wang, Junhui Shi, Junjie Yao, Lei Li, Ruiying Zhang, Chih-Hsien Huang, Jun Zou, Lihong V. Wang

**Affiliations:** 1 Optical Imaging Laboratory, Department of Biomedical Engineering, Washington University in St. Louis, St. Louis, MO 63130, USA; 2 Department of Electrical and Computer Engineering, Texas A&M University, College Station, TX 77843, USA

## Abstract

Metastasis causes as many as 90% of cancer-related deaths, especially for the deadliest skin cancer, melanoma. Since hematogenous dissemination of circulating tumor cells is the major route of metastasis, detection and destruction of circulating tumor cells are vital for impeding metastasis and improving patient prognosis. Exploiting the exquisite intrinsic optical absorption contrast of circulating melanoma cells, we developed dual-wavelength photoacoustic flow cytography coupled with a nanosecond-pulsed melanoma-specific laser therapy mechanism. We have successfully achieved *in vivo* label-free imaging of rare single circulating melanoma cells in both arteries and veins of mice. Further, the photoacoustic signal from a circulating melanoma cell immediately hardware-triggers a lethal pinpoint laser irradiation to kill it on the spot in a thermally confined manner without causing collateral damage. A pseudo-therapy study including both *in vivo* and *in vitro* experiments demonstrated the performance and the potential clinical value of our method, which can facilitate early treatment of metastasis by clearing circulating tumor cells from vasculature.

As many as 90% of cancer-related deaths are attributed to metastases, mostly taking the route of hematogenous dissemination of circulating tumor cells (CTCs)^1^,^2^,^3^,^4^. Melanoma has a high propensity for metastasis even at an early stage^5^,^6^, therefore early detection and treatment of melanoma CTCs are vital for reducing the risks of metastasis and improving patient prognosis^4^,^7^. Clinical *ex vivo* CTC detection with high sensitivity remains a challenge since it heavily depends on both small-volume sampling of blood and labeling of biomarkers on the cell membrane^3^,^4^,^8^,^9^. Extensive *in vivo* CTC detection studies employing fluorescence probes^9^,^10^ or multiplex nanoparticles^11^,^12^ have achieved promising results, but the biosafety and labeling efficiency remain critical concerns, which limits their clinical translation. Compared to other biological tissue components, melanoma cells have a high melanosome expression, providing a striking endogenous absorption contrast in the red to near-infrared (NIR) spectrum^13^,^14^. Hence, photoacoustic (PA) imaging, having the highest possible sensitivity to absorption contrast^15^,^16^,^17^, is a perfect tool for label-free imaging of melanoma cells^17^,^18^,^19^. In 2009, Galanzha *et al*. reported label-free detection of melanoma CTCs *in vivo* using PA cytometry^12^, in which a pulsed laser was focused across a blood vessel and an ultrasonic transducer collected PA signals from a large voxel. This design enables detection of melanoma CTCs *in situ*, but it lacks imaging capabilities. A recently developed optical-resolution PA imaging technique has acquired images of single CTCs *in vivo*^18^. However its non-optic-acoustic-confocal configuration and low CTC-to-background contrast limit the applications to capillaries and require complicated pattern recognition to identify CTCs^18^. High-contrast flow cytography that can reliably distinguish CTCs in real-time is needed for on-the-fly targeted CTC therapies.

Traditional clinical therapies for tumor metastasis are still palliative, with many drugs not reaching metastasis sites^2^,^20^. Novel therapies based on particular metastasis features, such as angiogenesis, lymphangiogenesis^21^, specific signal pathways and biomarkers^20^, have reported good clinical outcome, but they have less efficacy on initial micro-metastasis sites and typically produce adverse side effects^22^,^23^. Taking another approach, therapies directly targeting CTCs can reduce tumor metastasis potentials and may even prevent metastasis from occurring if administered in early tumor stages^4^,^7^. Nanosecond-pulsed lasers at 680–1064 nm wavelengths have long enabled physicians to treat melanocytic skin lesions with a high degree of selectivity and a low risk of postoperative complications^24^,^25^. For laser pulses shorter than the thermal relaxation time, radiant energy is largely confined in the absorber^26^, which can achieve efficient energy delivery and selective photothermolysis of the absorbing cells^27^. When a nanosecond laser pulse with sufficient energy irradiates a melanoma CTC, the melanosomes inside are heated beyond the threshold temperature for explosive vaporization, photomechanically killing the CTC without damaging any adjacent tissue components^26^,^27^.

Here we report high-resolution CTC imaging, using our new dual-wavelength PA flow cytography technology, in combination with real-time CTC destruction at the single-cell scale by pinpoint nanosecond-pulsed lethal irradiation from a therapy laser. We obtained images of single CTCs flowing in both arteries and veins on the fly, and performed real-time CTC destruction *in vivo* in small animals. The performance of this system was demonstrated by a study mimicking treatment of melanoma metastasis.

## Results

### Imaging results

**Figure 1:**
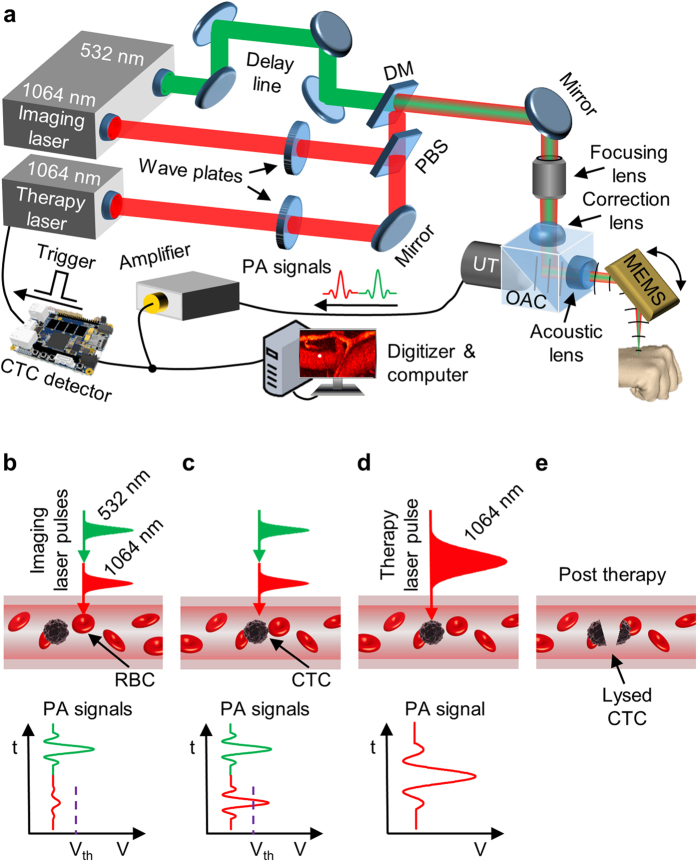
CTC imaging and destruction by dual-wavelength PA flow cytography combined with laser therapy. (**a**) Schematic of selected components of the experimental system. DM, dichroic mirror; MEMS, micro-electro-mechanical-system scanning mirror; OAC, optical-acoustic combiner; PBS, polarizing beamsplitter; UT, ultrasonic transducer. The 1064 nm and 532 nm imaging lasers are employed to image CTCs and vasculature, respectively. (**b–e**) Scheme of real-time detection and laser killing of CTCs. The CTC detector compares the earlier 1064 nm laser-induced CTC-specific PA signal against an optimized threshold level (purple dashed line in (**b**) and (**c**)) above the Hb signal, and thus can reliably distinguish CTCs and trigger the therapy laser (**c**). Within ~10 μs, the therapy laser is fired and focused to the detected CTC location to photomechanically kill the CTC (**e**).

**Figure 2:**
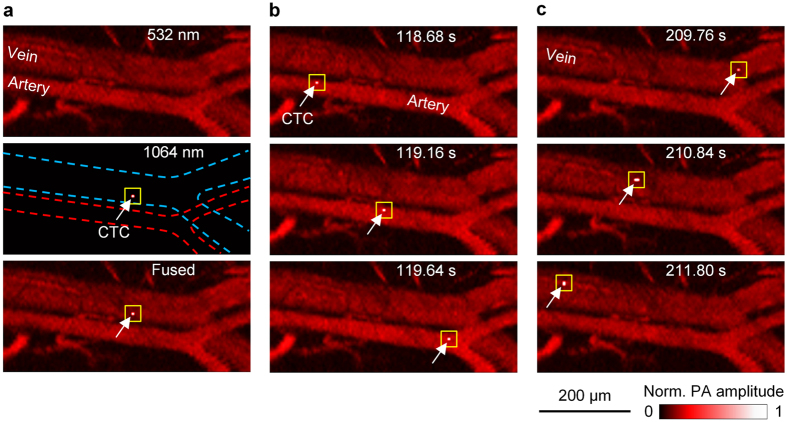
Snapshots showing single CTCs travelling in vasculature. (**a**) 532 nm laser-induced (Top), 1064 nm laser-induced (Middle), and fused (Bottom) flow cytography images. In the 1064 nm laser-induced image, the white arrow and yellow square indicate the detected CTC; the red and blue dashed lines delineate the artery and vein boundaries, respectively. (**b**) Three fused snapshots spanning ~1 s, showing a single CTC traveling in the artery. (**c**) Three fused snapshots spanning ~2 s, showing a single CTC traveling in the vein. The times labeled in (**b**) and (**c**) are relative to CTC injection.

To exploit the NIR absorption contrast between melanosome and hemoglobin (Hb) in red blood cells (RBCs), and to image CTCs on the fly, we developed a fast scanning PA flow cytography system for high-speed imaging at both 532 nm and 1064 nm wavelengths with single-cell resolution coupled with real-time selective CTC destruction by nanosecond-pulsed NIR laser-induced photothermolysis (Fig. 1, Methods). The therapy laser beam is coaxially aligned with the two imaging laser beams, and is triggered, using real-time hardware, immediately after flow cytography detects a CTC (Fig. 1b–e, Supplementary Fig. S1). We first validated the system’s ability to image single CTCs with the therapy laser turned off. To simulate CTCs, ~10^6^ cultured B16F10 melanoma cells were administrated into the mouse’s blood circulation system through jugular vein cannulation, and a portion of these cells survived in circulation as CTCs. A 1.2 × 0.3 mm^2^ area of an artery-vein pair in the mouse ear was imaged by flow cytography at a 16.6 Hz volumetric (3D) rate. Figure 2 shows some snapshots of single CTCs flowing through the field of view (FOV) in the artery and the vein, and Supplementary Movie S1 shows the entire time course. As shown in Fig. 2a, the vascular structure was imaged with high contrast by the 532 nm laser owing to the strong light absorption by Hb in RBCs at this wavelength. However, CTCs can hardly be distinguished from RBCs in these images, mainly due to the similar absorption coefficients for melanosome and Hb at 532 nm. In comparison, the 1064 nm laser pulse excitation of a CTC produced a PA signal with ~5 times greater amplitude than the background, while RBC signals were below the noise level. We performed control studies for 20 minutes before cell injection and recorded no PA signals of a similar amplitude level, which verified that the high-amplitude 1064 nm laser-induced PA signals indeed originated only from CTC absorption. The arterial and venous flow speeds of the CTC were calculated to be 0.33 mm/s and 0.20 mm/s, respectively, which are in general accordance with previously reported values^28^. A CTC cluster flowing in an artery was also imaged *in vivo* in a carotid artery cell injection experiment (Supplementary Fig. S2). No return of this CTC cluster was observed, which indicated that it might have been clogged at a vascular branching point, seeding a secondary tumor^10^.

### Therapy results

To investigate the radiant energy required to kill a CTC, we used a single-shot 1064 nm laser pulse to irradiate cultured B16F10 melanoma cells *in vitro*. Different areas of a monolayer of melanoma cells were irradiated with increasing levels of laser fluence, and then the cells were stained with trypan blue to test cell viability (Supplementary Fig. S3). The results indicated that a 1064 nm laser pulse with 8.8 J/cm^2^ fluence was sufficient to guarantee cell death. Potential tissue damage by the therapy laser was also carefully investigated. A monolayer of RBCs was imaged by phase contrast microscopy before and after 30 J/cm^2^ laser irradiation (Supplementary Fig. S4). Comparison of the two images indicated no apparent change, with both showing donut-shaped morphology. In addition, measuring the optical absorbance of the supernatant showed no RBC hemolysis in blood samples exposed to 30 J/cm^2^ laser irradiation (Supplementary Fig. S5).

**Figure 3:**
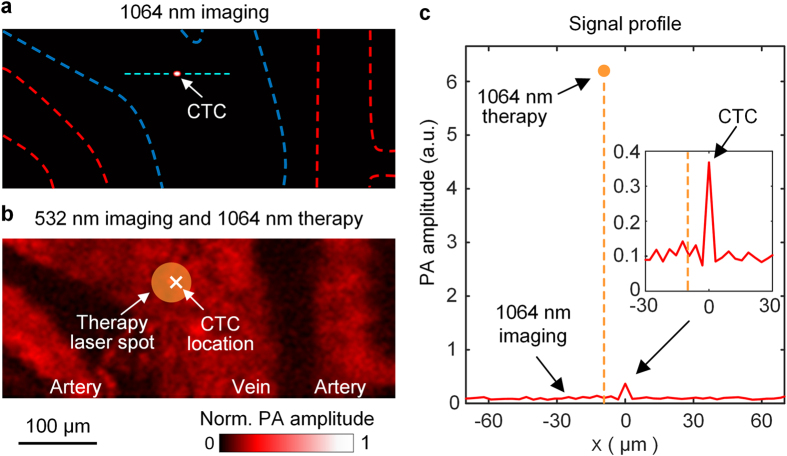
*In vivo* detection and expected photothermal killing of a CTC. The CTC was first detected in the 1064 nm laser-induced flow cytography image (**a**) and then lethally irradiated by a therapy laser pulse, also at 1064 nm, with a 50-μm focal diameter (**b**). In (**b**), The much-higher-amplitude PA signal induced by the therapy laser pulse is illustrated by the filled yellow circle. (**c**) Profile of the PA signals from the region across the CTC, indicated by the dashed cyan line in (**a**). The *x* axis, parallel with the imaging laser’s scanning direction (from right to left), was centered at the CTC location. In the 1064 nm imaging laser-induced signals (red solid line), the CTC signal (shown in detail by the inset) had a contrast-to-noise-ratio of ~25. The therapy laser-induced PA signal’s peak location (dashed orange line) was only ~10 μm away from the detected CTC location, which indicated that the CTC position (white cross in (**b**)) was within the therapy laser’s focal spot (circle in (**b**)).

Therapy experiments were conducted in a similar manner to the imaging experiments. Figure 3 shows an event of single-CTC detection and real-time destruction. The CTC signal induced by the 1064 nm laser pulse immediately hardware-triggered a therapy laser pulse to irradiate the detected CTC with a 50 μm focal diameter and a 25 J/cm^2^ fluence, above the lethal level. This lethal irradiation produced a PA signal with a much greater amplitude, ~14 times higher than the maximal imaging signal (Fig. 3c), and its peak location indicated that the CTC was within the focal spot of the therapy laser. This PA signal amplitude, measured in another experiment, was far higher than that of a regular blood vessel with the same irradiation (Supplementary Fig. S6). Therefore, we concluded that this PA signal mainly originated from light absorption by the CTC, and that this CTC was destroyed by the therapy laser irradiation. In total, four events of real-time expected photothermal killing of CTCs at different locations were recorded during 5 minutes in the experiment (Supplementary Movie S2–3). Flow cytography confirmed no disturbance to blood flow during 30 min of observation after this experiment. In addition, there were no visible damages on the mouse ear.

**Figure 4:**
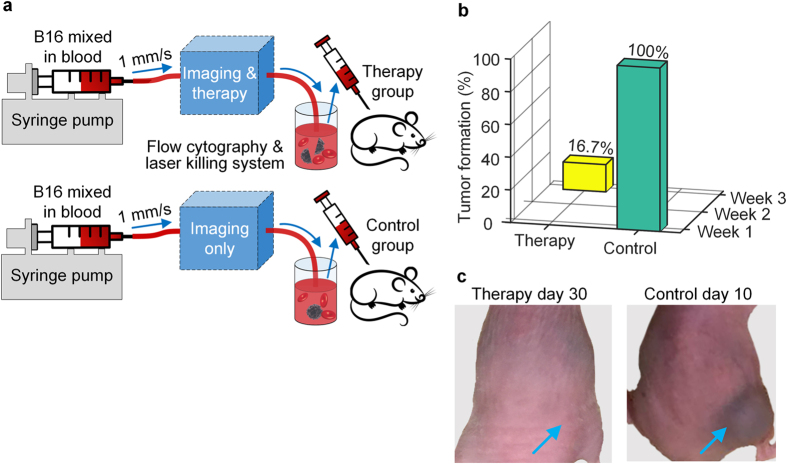
Scheme (**a**) and results (**b,c**) of the pseudo-therapy study. (**a**) The control experiment (bottom) was conducted after the therapy experiment (top) by simply switching off the therapy laser. The flow rate was set at ~80 μL/hr with ~20 CTCs flowing through the system every second. (**b**) Only 1 out of 6 therapy experiments had a tumor detected at week 3, compared to 100% tumor formation by week 1 in the control group. (**c**) Representative photos of the mice after experiments. (Left) No tumor was detected around the inoculation site (blue arrow) in 30 days following a therapy experiment. (Right) A raised tumor (blue arrow) was observed 10 days after a control experiment.

We also conducted a pseudo-therapy study to evaluate the performance of the system and demonstrate the value of this therapy scheme. Experiments were designed to mimic treating melanoma metastasis by impeding CTC dissemination (Fig. 4a). First, cultured B16F10 cells were mixed with bovine blood and pumped into a translucent silicone tube to simulate CTCs. Then this mixture was pumped through our system to specifically detect and kill the CTCs (Supplementary Movie S4). For the control experiment, the therapy laser was turned off during the experiment, leaving the CTCs only imaged by our system. Next, the treated and untreated mixtures were subcutaneously inoculated into two groups of nude mice, respectively, simulating hematogenous metastases. Afterwards, the area around each inoculation site was imaged every week by acoustic resolution (AR) PAM^17^,^29^ at 750 nm wavelength, to monitor tumor formation (Supplementary Fig. S7). After 30 days, only one of the six therapy experiments resulted in a tumor formation, and that tumor had a significantly slower growth, in comparison to the rapid tumor formation and growth following every control experiment (Fig. 4b,c, Supplementary Fig. S8). This only tumor from the therapy experiment could be attributed to the fact that the therapy laser (1 kHz maximum repetition rate) missed some CTCs that were detected within 1 ms after the last therapy laser shot (Supplementary Movie S4), which should not present a real problem since CTCs are more rare *in vivo* and the repetition rate of the therapy laser can be increased. The results indicated that our technique is capable of destroying CTCs with a high success rate, and that this therapy scheme is a promising way to impede metastasis for cancer therapy.

## Discussion

We report both *in vivo* label-free imaging of melanoma CTCs using dual-wavelength PA flow cytography and on-the-spot pinpoint CTC destruction by nanosecond-pulsed NIR therapy laser irradiation. CTCs are important indicators of the severity of a tumor and the efficacy of tumor therapies^1^,^2^,^3^,^5^,^8^, making their reliable detection clinically significant. Because some CTCs are cloaked by platelets or coagulation factors, they are shielded from many CTC detection agents as well as from the immune system^3^,^8^. However, our method does not suffer from this limitation because light can penetrate CTCs. This technique may be used clinically for non-invasive *in vivo* CTC assays without labeling. In addition, many details about the process of tumor metastasis through CTCs are still unclear, and this high-resolution real-time CTC imaging technique can enable *in vivo* studies of CTC dynamics, such as monitoring tumor cells shedding from a primary tumor, invading nearby blood vessels, circulating in the vasculature, and extravasating from vascular walls.

Unlike therapeutics that use metastasis-specific features to direct the distribution of administered medication, the reported method works on a more selective “identify-then-locally-administer” basis. Because the radiant energy from the therapy laser is highly localized on the melanosomes within CTCs, other molecules in the CTCs, as well as adjacent RBCs, do not sustain damage. Moreover, our technology may have extra benefits in immunotherapy because it can help release viable tumor-specific antigens from lysed CTCs into vasculature, which can stimulate immune attacks on remaining CTCs as well as metastases, enhancing treatment efficacy^30^. Drug resistance from CTCs is also not an issue in our study because the destruction mechanism is completely physical.

Melanoma patients at all stages would potentially benefit from this technology if it were translated into the clinic successfully. For stages I and II, measuring CTC count could yield a more accurate diagnosis of a melanoma’s recurrence risks. For stage III, when there are CTCs in the bloodstream but no existing metastases, and stage IV, when there are CTCs in the bloodstream and existing metastases, the clearance of CTCs would potentially prevent metastasis. The immunoresponses induced by antigens released by lysed CTCs could potentially further treat any residual primary tumor as well as existing metastases.

In summary, using endogenous contrast, our system dynamically imaged rare single CTCs and CTC clusters with single-cell resolution *in vivo* and performed real-time pinpoint photothermolysis of CTCs. The pseudo-therapy experiment demonstrated that our method can effectively kill CTCs in vasculature. This technology works in a convenient reflection mode, and is translatable to clinics. It can serve as a bedside therapy device for cancer patients, which can improve their prognosis by early detection and destruction of CTCs. The technology can also be applied for studying fundamental mechanisms of tumor metastasis through CTC dissemination.

## Methods

All methods were performed in accordance with the guidelines of Washington University in St. Louis.

### Dual-wavelength PA flow cytography

To induce PA signals, we employed a high-repetition-rate picosecond laser (APL-4000–1064, Attodyne, Inc.; maximum pulse repetition rate: 500 kHz) to provide 6-ps laser pulses at 1064 nm and 532 nm wavelengths. After traveling a 25-meter delay line, the 532 nm laser beam was combined with the 1064 nm laser beam through a longpass dichroic mirror. Melanosome absorbs similarly to Hb at 532 nm, but >10 times more strongly than Hb at 1064 nm, which provides a high CTC contrast against the RBC background. The combined laser beams were focused by a focusing lens, reflected by an optical-acoustic combiner (OAC), and directed to the target by a water-immersible MEMS (micro-electro-mechanical system) mirror for 1D fast scanning^31^,^32^. The target was mounted on a stepper-motor-driven translational stage to facilitate orthogonal slow axis scanning. The OAC comprised of an aluminum-coated prism and an uncoated prism, and the thin aluminum coating on the first prism reflected light but transmitted sound. A correction lens was attached to the top surface of the OAC to correct the optical aberration due to the prisms. Excited PA waves were also reflected by this MEMS mirror, collected by an acoustic lens, transmitted through the OAC, and detected by an ultrasonic transducer (V214-BB-RM, Olympus-NDT, Inc.). Since the 532 nm laser pulse traveled through the ~80-ns delay line, the PA wave induced by the 532 nm laser pulse arrived ~80 ns later than that by the 1064 nm laser pulse (Fig. 1a). PA signals were then amplified by two radio-frequency amplifiers (ZX60-3018G-S+ and ZFL-500LN+, Mini-circuits, Inc.) and acquired by a high-speed digitizer (ATS9350, Alazar Tech, Inc.). By steering both the optical and acoustic axes simultaneously, the system maintained confocal alignment over the entire FOV, providing high detection sensitivity. This flow cytography was capable of volumetric (3D) imaging at 10 Hz over a FOV of 3 × 0.25 mm^2^ at a depth up to ~0.7 mm, with a 3 μm lateral resolution for the 532 nm flow cytography images, 7 μm for the 1064 nm flow cytography images, and ~26 μm axial (depth) resolution for both. The length and width of the FOV can be tuned by varying the driving voltage of the MEMS mirror and by adjusting the scanning range of the stepper motor, respectively. It is also possible to achieve a higher frame rate at the expense of a larger scanning step size, as in Fig. 2, or of a shorter range for the stepper motor.

### On-the-fly CTC detection

PA signals were concurrently analyzed for melanoma CTC detection based on the melanosome-specific 1064 nm absorption induced PA signals (Supplementary Fig. S1). The specially built CTC detector consisted of an ultrafast analog switch and a comparator (Supplementary Fig. S1). Based on the fixed 80-ns time interval between the 1064 nm and 532 nm laser pulses, a time window signal controlled the analog switch as a gate to selectively transmit the earlier 1064 nm laser-induced PA signal. The comparator compared this gated PA signal with an optimized preset threshold voltage. A signal above the threshold indicated the presence of a melanoma CTC, and immediately triggered a high-pulse-energy therapy laser (INNOSLAB, EdgeWave, Inc.) to lethally irradiate the same CTC on the spot (Fig. 1c,d). Following a successful CTC detection, the detector was disabled for 1 ms to prevent double triggering by the much-higher-amplitude therapy laser-induced PA signal.

### Real-time CTC destruction

Melanoma CTCs were irradiated by a high-pulse-energy 1064 nm laser pulse (7 ns) that could mechanically destroy the CTCs by explosive vaporization of the melanosomes inside^26^,^27^,^33^. The thermal diffusion distance during 7-ns laser exposure is ~30 nm, which means the thermal damage is confined to melanosomes, ~500 nm in diameter^27^. The therapy laser beam was coaxially aligned with the imaging laser beams by a polarizing beamsplitter and focused onto the CTC location. In order to maximize combining efficiency, a half-wave plate was used to adjust the polarization of the therapy laser beam. We optimized the focal spot size of the therapy laser based on the scanning speed of the MEMS mirror, the signal propagation and processing time, the CTC flow speed, and the therapy laser’s trigger-to-emission delay. The maximum scanning speed of the laser focal spot on the target was ~1 m/s. The acoustic flight time from the absorber to the ultrasonic transducer was ~10 μs, while the signal processing time in the CTC detector circuits was ~30 ns and the trigger-delay time of the therapy laser was ~300 ns. Based on the blood flow speed, the CTC movement in blood vessel during the ~10 μs response time was negligible. In conclusion, the center of the therapy laser’s focal spot could be ~10 μm (1 m/s × 10 μs) away from the original imaged CTC location. Therefore, the focal spot of the therapy laser, with 25 J/cm^2^ fluence, was adjusted to be 50 μm in diameter to ensure coverage of the entire CTC with a sufficient local radiant intensity.

### Experimental animals

Adult female ND4 Swiss Webster mice (Hsd: ND4, Envigo, Inc.; 20–25 g, 10–12 weeks old) were used for the *in vivo* CTC imaging and laser killing experiment. The laboratory animal protocols were approved by the Animal Studies Committee of Washington University in St. Louis. Three days before the experiment, cannulation was performed to safely insert a catheter into the jugular vein or carotid artery of the mouse, and the hair on the mouse ear was removed with human hair-removing lotion. During the experiment, the mouse was maintained under anesthesia with 1.5% vaporized isoflurane, and taped to a lab-made animal holder, which was mounted on the stepper-motor-driven translation stage. Ultrasound gel was then applied to the imaging area to retain moisture and couple acoustic signals. A water tank filled with deionized water was then placed on top of the mouse ear. The membrane at the bottom of the water tank was in gentle contact with the ultrasound gel. During imaging, a 0.1-ml suspension containing ~10^6^ B16F10 melanoma cells was injected through the cannulation catheter. The cells were cultured in DMEM supplemented with 10% fetal bovine serum at 37 °C in a humidified atmosphere with 5% CO_2_.

Female athymic nude mice (Hsd:Athymic Nude-Foxn1, Envigo, Inc.; 12–15 g, 3–4 weeks old) were used for the pseudo-therapy study. The laboratory animal protocols were approved by the Animal Studies Committee of Washington University in St. Louis. Before the study, the mice were randomly assigned into either the therapy group or the control group. In one experiment, a mixture was first prepared by diffusing cultured B16F10 melanoma cells into bovine blood (Defibrinated Bovine Blood, Quad Five, Inc.). Next, this mixture was pumped in a translucent silicone tube (300 μm inner diameter and 640 μm outer diameter)—mimicking a blood vessel—through our system with the therapy laser turned on. The flow rate was set at ~80 μL/hr, with ~20 CTCs passing through the system every second. Afterwards, the cell mixture was reduced in volume by centrifugation to make a 50 μL dose (containing ~10^5^ cells) that was subcutaneously inoculated into the dorsal area of a nude mouse in the therapy group. Then, another vial of mixture was acquired in a similar manner but with the therapy laser turned off, and a 50 μL dose of this imaged (but not treated) mixture was inoculated into a nude mouse in the control group. Afterwards, all the mice were imaged by AR-PAM to detect tumor formation and to measure the volume of flat tumors, and raised tumors were measured using a caliper^34^. Mice were euthanized when the tumor dimension exceeded 2 cm or the tumor began to ulcerate.

## Additional Information

**How to cite this article:** He, Y. *et al*. *In vivo* label-free photoacoustic flow cytography and on-the-spot laser killing of single circulating melanoma cells. *Sci. Rep.* **6**, 39616; doi: 10.1038/srep39616 (2016).

**Publisher's note:** Springer Nature remains neutral with regard to jurisdictional claims in published maps and institutional affiliations.

## Supplementary Material

Supplementary Movie S1

Supplementary Movie S2

Supplementary Movie S3

Supplementary Movie S4

Supplementary Information
