# Supplementary material for: In vivo label-free photoacoustic flow cytography and on-the-spot laser killing of single circulating melanoma cells
Source: Sci Rep. 2016 Dec 21;6:39616. doi: 10.1038/srep39616 (PMC5175175; doi:10.1038/srep39616)
Supplement: Supplementary Information [file srep39616-s5.pdf]

In vivo label-free photoacoustic flow cytography and on-the-spot laser killing of single circulating melanoma cells

Yun He[1, ‡], Lidai Wang[1, ‡, †], Junhui Shi[1, ‡], Junjie Yao[1], Lei Li[1], Ruiying Zhang[1], Chih-Hsien Huang[2], Jun Zou[2], Lihong V. Wang[1, *]

[1]Optical Imaging Laboratory, Department of Biomedical Engineering, Washington University in St. Louis, St. Louis, MO 63130, USA.

[†]Current affiliation: Department of Mechanical and Biomedical Engineering, City University of Hong Kong, Kowloon, Hong Kong, China.

[2]Department of Electrical and Computer Engineering, Texas A&M University, College Station, TX 77843, USA.

[‡]These authors contributed equally to this work.

*Corresponding author. E-mail: lhwang@wustl.edu

Supplementary Figures

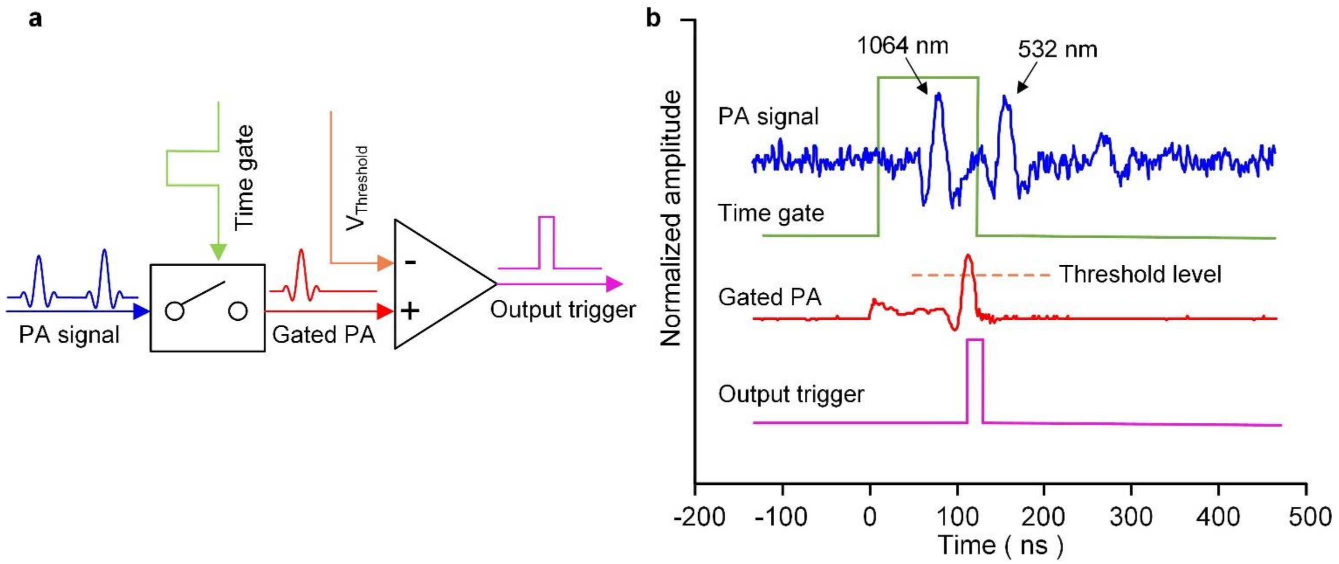

Supplementary Figure S1. Circuit diagram and performance of the lab-made CTC detector. (**a**) Circuit diagram. The time gate signal (TTL logic) controls an ultrafast analog switch (SN74LVC1G3157, Texas Instruments Inc.) as a gate. Based on the fixed 80-ns time interval between the 1064 nm and 532 nm laser pulses in the dual-wavelength flow cytography, the time gate signal is employed to selectively gate the PA signals, allowing only the earlier PA signal induced by the 1064 nm laser pulse to pass through to the comparator. The voltage comparator (TLV3502, Texas Instruments Inc.; 4.5 ns propagation time) compares the gated PA signal to distinguish CTCs from RBCs and other blood cells. Once the signal peak is above the optimized preset threshold voltage (80 mV), a trigger signal is generated for the therapy laser. The high-speed characteristics of the analog switch and comparator guarantee real-time CTC detection and therapy-laser triggering. (**b**) Time sequence plot of the PA signal, time gate control signal, gated PA signal, and output trigger. The time interval from the 1064 nm laser-induced PA signal from a CTC to the output trigger is ~30 ns.

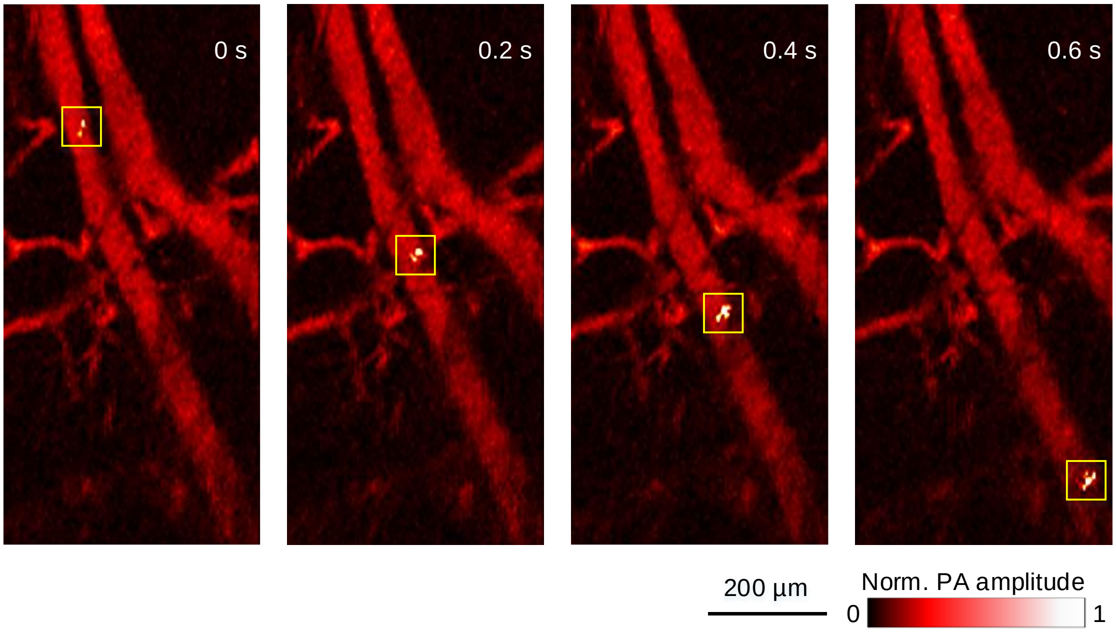

Supplementary Figure S2**. Sequential flow cytography images of a CTC cluster flowing through an artery in a mouse ear.** About 10^6 cultured B16F10 cells were administered through carotid artery cannulation. Some of the cells were naturally bound together by cell adhesion as clusters. A 2.0 × 0.5 mm^2 area of vasculature over a depth of ~150 µm in the mouse ear was imaged by flow cytography at a 5 Hz volumetric (3D) rate. As it flowed through the FOV, a CTC cluster in the artery was imaged in four successive frames. The size of the cluster was estimated to be 25-40 µm. Since the dynamics of CTC clusters may be turbulent inside the blood vessel and the scanning step size is ~5 µm, the PA signal of the CTC cluster fluctuated.

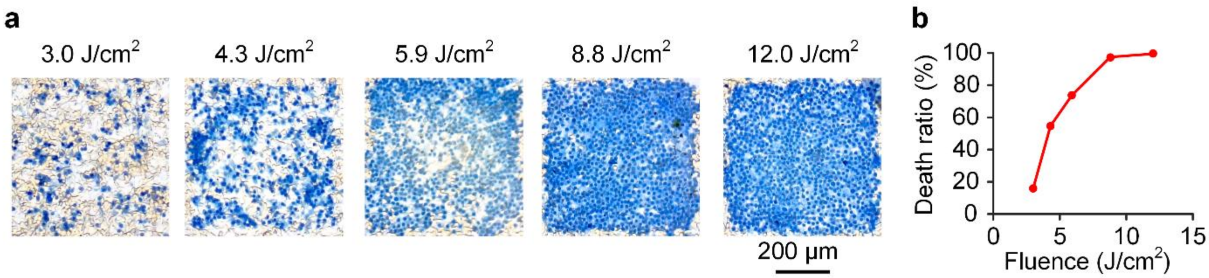

Supplementary Figure S**3. Irradiation of melanoma cells *in vitro***. (**a**) B16 cells on glass slides were irradiated by increasing levels of laser fluence and stained with trypan blue to test cell viability. (**b**) Plot of cell death percentage versus laser fluence.

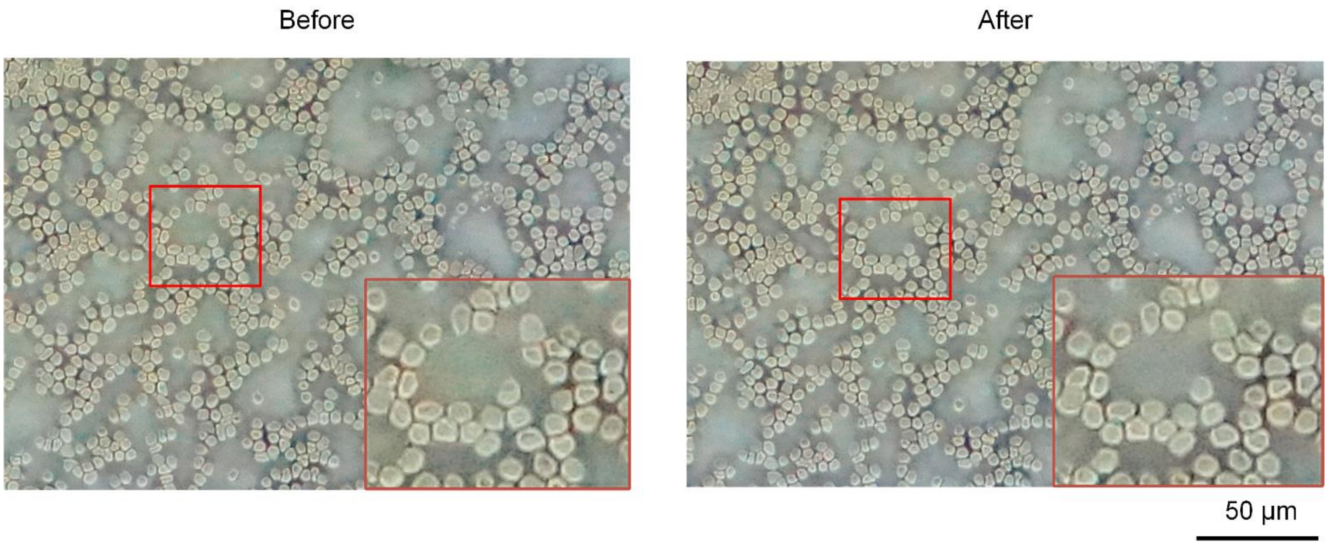

Supplementary Figure S**4. Phase-contrast images of RBCs acquired before and after exposure to single-shot 1064 nm laser irradiation with 30 J/cm^2 fluence.** Close-ups of the same region (enclosed by the red rectangles) before and after exposure, placed at the bottom right corners of the two images, indicate no apparent morphological change.

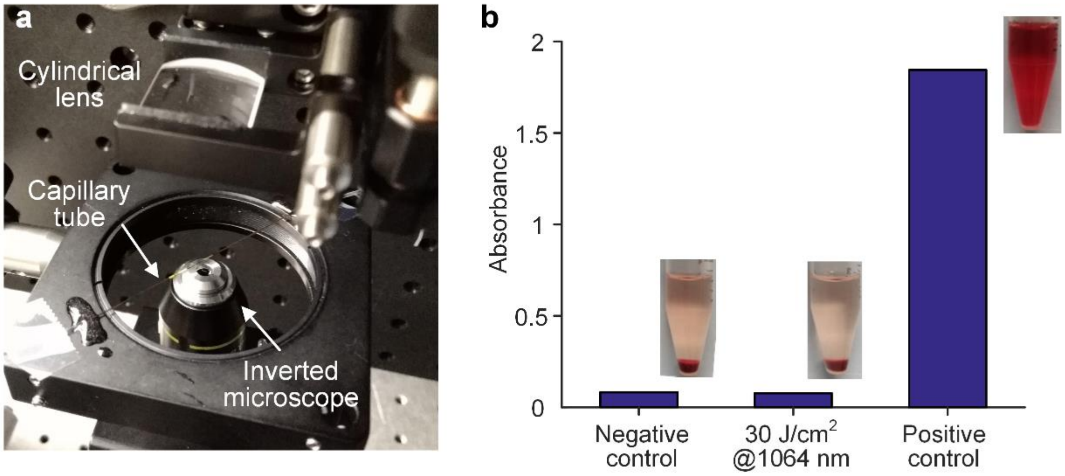

Supplementary Figure S5. RBC hemolysis study results. (**a**) Potential RBC hemolysis by an energetic 1064 nm laser was studied by irradiating flowing blood samples at a 30 J/cm^2 fluence level. Bovine blood was washed three times with saline before the experiment, then it was pumped through a glass tube (100 µm inner diameter) at a 0.15 mm/s flow speed. Laser light (5 kHz repetition rate) was focused across the tube by a cylindrical lens with a 30 µm focal width. An inverted microscope was used to help position the tube in the focal zone of the lens. (**b**) The optical absorbance of the supernatants at 532 nm indicates no detectable hemolysis was caused by the therapy laser irradiation. For the two control groups, the therapy laser was turned off during pumping. Blood samples from the negative control and the study groups were diluted 20 times with saline, while that of the positive control group was diluted 20 times with pure water to induce total hemolysis. All blood samples are centrifuged at 3000 rpm for 10 min, and the supernatants (photos shown at the top right corner of each group) were extracted to measure their optical absorbance at 532 nm. Equation $R = (A_s - A_0)/A_{100}$ is used to calculate the hemolysis ratio R[35], where A_s is the absorbance of the supernatant from the study group, and A_0, and A_{100} are that from blood samples with no hemolysis (negative control) and 100% hemolysis (positive control), respectively.

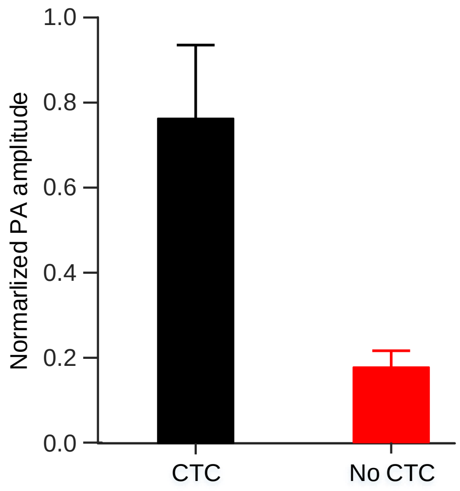

Supplementary Figure S6. Therapy laser-induced PA signals. Therapy laser-induced PA signals from a blood vessel containing a CTC within the laser's focal spot (black), and that from the same blood vessel when it does not contain a CTC (red). Data are averaged over ten measurements; error bars show the standard deviations.

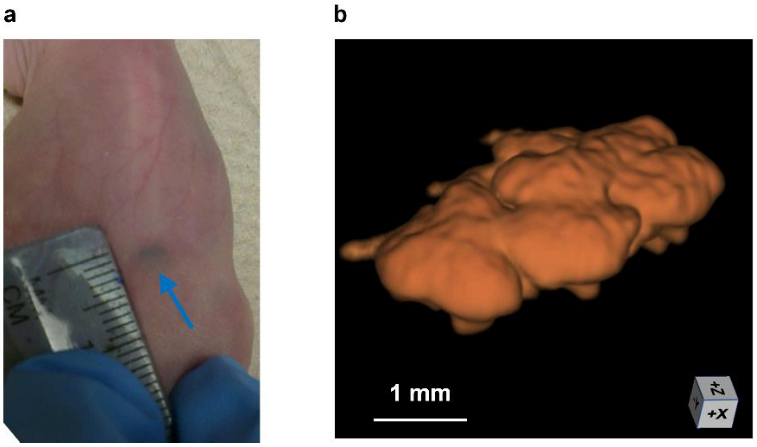

Supplementary Figure S**7. Tumor detection by visual observation and by AR-PAM.** (**a**) Photograph of a flat tumor (blue arrow) in the control group taken 5 days after experiment. (**b**) Three-dimensional AR-PAM image of this tumor. Its volume is calculated to be 2.01 mm^3.

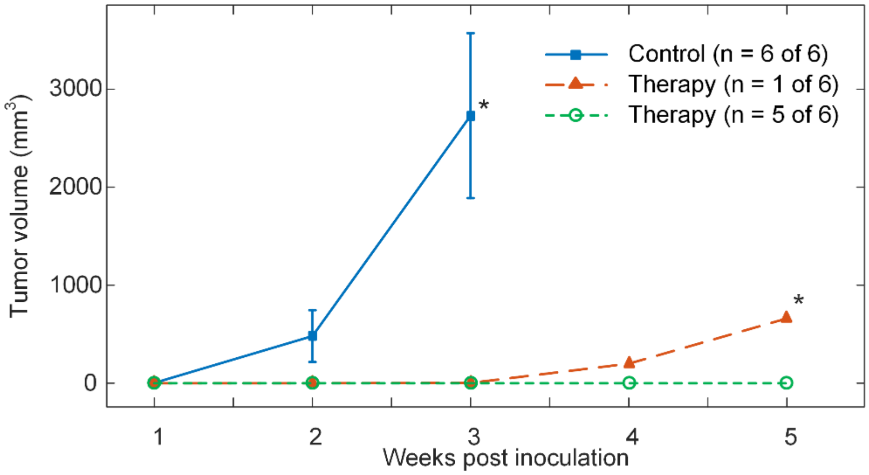

Supplementary Figure S**8. Comparison of tumor growth in the pseudo-therapy study.** No tumor was detected using AR-PAM from five out of six therapy experiments by week 5 (green dashed line), and the only other therapy experiment resulted with a tumor that had a significantly slower growth (red dashed line), compared with that of the control experiments (blue solid line). Tumor volume was measured by AR-PAM (for flat tumors) or estimated by the formula $\pi/6 \times L \times W \times H$ (for raised tumors), where L, W, and H represent length, width, and height, respectively. Asterisk: mouse euthanasia; error bar: standard deviation.

Reference:

35. Krzyzaniak, J. F., Raymond, D. M. & Yalkowsky, S. H. Lysis of human red blood cells 1: effect of contact time on water induced hemolysis. PDA Journal of Pharmaceutical Science and Technology 50, 223-226 (1996).

Supplementary Movie Captions

Supplementary Movie S1.

Video of single CTCs flowing in an artery-vein pair in a mouse ear. A 1.2 × 0.3 mm^2 area of a mouse ear containing an artery-vein pair was imaged by the dual-wavelength PA flow cytography. The video is played in real time at 16.6 Hz. (Top) Composed of flow cytography images induced by 532 nm laser pulses, the video shows blood vessel structures clearly. The upper major vessel is a vein, while the lower major vessel is an artery. (Middle) Composed of flow cytography images induced by 1064 nm laser pulses, the video reveals melanoma CTCs, which were detected with high contrast. The imaged CTCs are indicated by yellow square boxes. The blood vessel boundaries are sketched in dashed lines (red for arteries, blue for veins), found by averaging all of the frames in the movie. (Bottom). The video is produced by fusing the top and middle flow cytography images.

Supplementary Movie S2.

Video of on-the-spot destruction of single CTCs in a mouse ear. The 1064 nm imaging-laser-induced PA signals from CTCs were detected and then used immediately to trigger the therapy laser (also at 1064 nm). The videos are composed of fused PA images from the imaging lasers and the therapy laser. (Top, left column) Video from a control experiment performed prior to the CTC injection. The whole control experiment lasted for 20 minutes, and recorded no 1064 nm imaging-laser-induced PA signals with the amplitude expected from a CTC. Here, we show only 8 seconds for comparison. (Bottom, left column) Video acquired after CTCs were injected into the blood circulation. The white spots are CTCs detected from the 1064 nm laser-induced PA signals. A high-pulse-energy 1064 nm therapy laser pulse was triggered within ~10 μs and focused onto a 50-μm-diameter area containing the CTC (illustrated by a

yellow filled circle), killing it instantly. Four events of real-time photothermal destruction of single CTCs were recorded over 5 minutes and were are concatenated to produce this 7.1-second video. (Right column) Snapshots of moments when a therapy laser pulse was focused on a CTC.

Supplementary Movie S**3.**

Video showing the same contents as **Supplementary Movie** S**2,** except that, for easy observation, the video is paused each time a CTC is detected.

Supplementary Movie S**4.**

Video of the pseudo-therapy study showing CTCs pumped through the CTC flow cytography and laser killing system in a therapy and a control experiment. The videos are composed of fused PA images from the imaging lasers and the therapy laser. (Top) Video from a control experiment, CTCs are labeled with yellow squares. (Bottom) Video from a therapy experiment, the much-higher-amplitude PA signals induced by therapy laser shots (50 µm focal spot size) are illustrated with filled yellow circles. Videos were acquired at 8 Hz volumetric rate, and the flow speed of the liquid mixture was set as 1 mm/s, allowing CTCs to be detected at least once in the FOV. In the therapy experiment, about 94.5% of the CTCs were successfully shot by the therapy laser, limited only by the slow repetition rate of the therapy laser. Each experiment lasted 3 hours, and we show only 15 seconds here for demonstration.
